# Associations between metabolic disorders and risk of cancer in Danish men and women – a nationwide cohort study

**DOI:** 10.1186/s12885-016-2122-7

**Published:** 2016-02-22

**Authors:** Siv Mari Berger, Gunnar Gislason, Lynn L. Moore, Charlotte Andersson, Christian Torp-Pedersen, Gerald V. Denis, Michelle Dalgas Schmiegelow

**Affiliations:** Department of Cardiology, Herlev and Gentofte University Hospital, Hjertemedicinsk Forskning 1, post 635, Kildegårdsvej 28, opg. 8, 3. tv, 2900, Hellerup, Copenhagen, Denmark; Department of Health, Science and Technology, Aalborg University, Aalborg, Denmark; Department of Medicine, Boston University School of Medicine, Boston, MA USA; Department of Pharmacology and Experimental Therapeutics, Boston University School of Medicine, Boston, MA USA; National Institute of Public Health, University of Southern Denmark, Copenhagen, Denmark; The Danish Heart Foundation, Copenhagen, Denmark

**Keywords:** Cancer, Metabolic, Diabetes, Hypertension, Hypercholesterolemia, Epidemiology

## Abstract

**Background:**

The prevalence of metabolic disorders is increasing and has been suggested to increase cancer risk, but the relation between metabolic disorders and risk of cancer is unclear, especially in young adults. We investigated the associations between diabetes, hypertension, and hypercholesterolemia on risk of all-site as well as site-specific cancers.

**Methods:**

We consecutively included men and women from nationwide Danish registries 1996–2011, if age 20–89 and without cancer prior to date of entry. We followed them throughout 2012. Metabolic disorders were defined using discharge diagnosis codes and claimed prescriptions. We used time-dependent sex-stratified Poisson regression models adjusted for age and calendar year to assess associations between metabolic disorders, and risk of all-site and site-specific cancer (no metabolic disorders as reference).

**Results:**

Over a mean follow-up of 12.6 (±5.7 standard deviations [SD]) years, 4,826,142 individuals (50.2 % women) with a mean age of 41.4 (±18.9 SD) years had 423,942 incident cancers. Incidence rate ratios (IRRs) of all-site cancer in patients with diabetes or hypertension were highest immediately following diagnosis of metabolic disorder. In women, cancer risk associated with diabetes continued to decline albeit remained significant (IRRs of 1.18–1.22 in years 1–8 following diagnosis). For diabetes in men, and hypertension, IRRs stabilized and remained significantly increased after about one year with IRRs of 1.10-1.13 in men for diabetes, and 1.07–1.14 for hypertension in both sexes. Conversely, no association was observed between hypercholesterolemia (treatment with statins) and cancer risk. The association between hypertension and cancer risk was strongest in young adults aged 20–34 and decreased with advancing age.

**Conclusions:**

Diabetes and hypertension were associated with increased risk of all-site cancer.

**Electronic supplementary material:**

The online version of this article (doi:10.1186/s12885-016-2122-7) contains supplementary material, which is available to authorized users.

## Background

Cancer is among the leading causes of death across all age groups in the western world [1], and as the only European country, cancer is now the leading cause of death in Denmark in both men and women [2]. In parallel to the rising clinical, social and economic burdens of cancer [3, 4], the prevalences of associated metabolic disorders such as diabetes, hypertension and hypercholesterolemia are rapidly increasing [5], and the studies of the relation between metabolic disorders and cancer risk are conflicting. Overweight and obesity have been linked with excess cancer risk in numerous studies [6–8], although it may be, as with cardiovascular disease [9], that the metabolic disorders associated with obesity are stronger predictors of cancer risk than obesity itself [10, 11]. Greater attention has been paid to the possible linkage between metabolic disorders and cancer risk in recent years [12–14], but many smaller studies were limited by insufficient statistical power to study the associations between individual metabolic disorders and cancer risk across sex and the life span of adult life, especially for cancer subtypes. Although meta-analyses and large studies exist within the literature, with the metabolic syndrome and cancer (Me-Can) project as one of the largest with over 500,000 participants, large-scale studies investigating the associations between metabolic disorders, and risk of all-site cancer as well as site-specific cancers, are still sparse, and the association largely unaccounted for in young adults.

This nationwide cohort study aimed to investigate the associations between metabolic disorders, namely diabetes, hypertension and hypercholesterolemia, and risk of cancer (all sites) as well as selected cancer subtypes in men and women aged 20–89 years over 17 years of follow-up.

## Methods

### Data sources

In Denmark, the health care system is governmentally financed, and for administrative purposes the Danish government keeps comprehensive and nationwide registers on several health care and population related variables. Each resident is given a unique and permanent identification number; therefore, cross-linkage of the different national registries at an individual level is possible. For this study, we cross-linked data from four different registers. *The National Population Register* includes information on vital status, date of birth, and immigration/emigration. *The Danish National Patient Register* (DNPR) holds information on all hospitalizations since 1977, including dates and discharge diagnoses according to the International Classification of Diseases (ICD); ICD-8 was used until 1993 and ICD-10 from 1994 onwards. *The Danish Register of Causes of Death* holds information on diagnoses related to the cause of death. Finally, *the Danish Register of Medicinal Product Statistics* (National Prescription Register) keeps records of all prescriptions dispensed from Danish pharmacies since 1995; all drugs are coded according to the Anatomical Therapeutic Chemical (ATC) classification system, and the register has been found to be accurate [15].

### Study population

The study population of interest consisted of all Danish residents, included consecutively during January 1, 1996 through December 31, 2011. Each individual was included in the study population at the last of the following events: on January 1 1996, the date the individual turned 20 years old or the date of immigration to Denmark. The population was followed from date of entry until first cancer event, emigration, death, date of 90th birthday or December 31, 2012, whichever occurred first. We excluded individuals with a history of cancer (ICD-8 codes starting with numbers 140–209, ICD-10 codes C) prior to date of entry.

Non-melanoma skin cancer was not included in the definition of cancer (ICD-8 code 173, ICD-10 code C44), as these common and generally non-fatal cancers are most often diagnosed and treated by general practitioners, from whom data are not included in the Danish registers.

### Outcomes

Our primary outcome was first occurrence of any incident cancer (ICD-10 codes C). Our secondary outcomes were specific cancer types: breast (ICD-10 code C50), ovarian (ICD-10 code C56), endometrial (ICD-10 codes C54, C55), cervical (ICD-10 code C53), kidney (ICD-10 code C64), lower urinary tract (LUT; ICD-10 codes C66–C68), pancreatic, (ICD-10 code C25), hepatic (ICD-10 code C22), gall bladder (ICD-10 code C23), colorectal (ICD-10 code C18–C20), prostate (ICD-10 code C61), esophageal (ICD-10 code C15), and lung cancer (ICD-10 code C33, C34). In this study, we identified cancer events from the DNPR and the Danish Register of Causes of Death. A recent validation study reported that ICD-10 codes of cancer registered in the DNPR have a positive predictive value of 98.0 − 100 % [16].

### Definitions

The metabolic disorders of interest, *diabetes, hypertension* and *hypercholesterolemia*, were defined using ICD codes and claimed prescriptions from nationwide registers. Diabetes was defined as diabetes requiring glucose-lowering medication. Since statin is the drug of choice when initiating treatment of uncomplicated hypercholesterolemia or prophylactic in e.g. cardiovascular disorders or diabetes, we defined hypercholesterolemia as two claimed prescriptions for statins. In contrast, other lipid-lowering drugs, e.g. fibrates, as the first drug are more likely prescribed to patients with severe hypertriglyceridemia. Diabetes and hypercholesterolemia were thus defined as claiming two prescriptions of glucose-lowering medication (ATC code A10), and statins (ATC code C10AA) respectively (date of diagnosis as date of the second claimed prescription). Hypertension was defined as either 1) a diagnosis of hypertension (ICD-10 codes I10-I15) followed by a subsequent prescription claim for an antihypertensive drug within 90 days, or 2) as claimed prescriptions for two different classes of antihypertensive drugs, as described in details previously [17] (Additional file 1). We defined prevalent diabetes and hypercholesterolemia as fulfilling the definition for the respective disorder prior to study entry.

### Statistics

All metabolic disorders were modelled as time-dependent exposure variables. Each individual contributed with disease-free exposure time until date of diagnosis of a disorder, and from this day onwards with time exposed for the disorder. The lexis-macro was used for all analyses and several time-scales were used, i.e. calendar year (bands were split in 1-year intervals since January 1, 1996), and duration of each metabolic disorder (bands were split at the defined date of diagnosis and 3, 6, 9, 12, 18, 24 months and every third year hereafter). Dichotomous variables were then created for each metabolic disorder (e.g. diabetes first 3 months yes/no using the left end point as reference). Age was calculated at the beginning of each interval and rounded in 2-year age intervals. In all analyses, being without the metabolic disorder of interest was used as reference (e.g. diabetes in month 9–12 was compared with no diabetes).

Associations between metabolic disorders and cancer were assessed using multivariable Poisson regression models with the different metabolic disorders (diabetes, hypertension and hypercholesterolemia) included in the same model. We conducted predefined interaction analyses, specifically assessed interaction between each metabolic disorder, and age, calendar year, sex and duration of the metabolic disorder with all interaction analyses included in the same model. We assessed the cancer risk stratified by four pre-defined age-categories, i.e. 20– < 35, 35– < 50, 50– < 65 and ≥65 years.

All statistical calculations were performed using SAS, version 9.4® (SAS Institute Inc, Cary, NC).

### Other analyses

Metformin is sometimes prescribed to women of fertile age due to polycystic ovary syndrome. We therefore conducted a sensitivity analysis in which we excluded all prescriptions of metformin (ATC-code A10BA; metformin is the only available biguanid in Denmark) to women between 20 and 39 years of age [18].

Data on life style habits are not available in the administrative registries, but are correlated with socioeconomic status [19]. We therefore explored confounding by socioeconomic status in an analysis stratified by sex and adjusted for age, calendar year and “highest attained educational level” at study entry. Immigrants were excluded from the population for this sensitivity analysis, because educational level attained abroad is not registered in Danish registers.

### Ethics

In Denmark, no ethics approval is needed for retrospective register-based studies. This study was approved by the Danish Data Protection Agency (j.nr.: 2007-58-0015/GEH-2014016 I-Suite nr: 02734).

## Results

From the population register we identified 5,324,572 men and women aged 20–89 years during 1996–2011, and excluded non-resident individuals (*n* = 369,400), individuals with a history of cancer prior to date of entry (*n* = 129,028), and misregistered cancers (*n* = 2). The final study population consisted of 4,826,142 (49.8 % men and 50.2 % women). Over a mean follow-up of 12.6 years, there were a total of 423,942 incident cancers, corresponding to 30,708,457 person-years (*n* cancers = 216,806) for women and 30,282,029 person-years (*n* cancers = 207,136) for men (Table 1). Presence of metabolic disorders was rare in both women and men at study entry (Additional file 1: Table S1). At the end of follow-up, the total numbers of individuals with the disorder either at date of entry or developed during follow-up, were for diabetes 6.7 % of men and 5.6 % of women, for hypertension 22.0 % of men and 24.6 % of women, and for hypercholesterolemia 15.1 % of men and 13.6 % of women. Since risk of cancer varied according to time elapsed since diagnosis (P for all interactions < 0.001), cancer risk was assessed according to duration of the specific metabolic disorder. Additionally, due to significant interaction between hypertension and sex (P for interaction <0.001) and dyslipidemia and sex (P for interaction < 0.001), albeit not between diabetes and sex (P for interaction = 0.39), we stratified all analyses by sex.

**Table 1:** Exposure time in person years for selected conditions and their combinations in a population of 4,826,142 individuals

Condition	Women (N cancer)		Men (N cancer)	
Sex	30,708,457.18	(216,806)	30,282,029.28	(207,136)
Age				
20– < 30 years old	4,623,447.19	(2,408)	4,889,888.43	(2,353)
30– < 40 years old	5,623,447.19	(7,375)	5,942,332.42	(4,645)
40– < 50 years old	5,710,818.95	(18,630)	5,971,215.73	(9,673)
50– < 60 years old	5,450,322.94	(38,244)	5,605,080.44	(28,144)
60– < 70 years old	4,376,820.30	(54,142)	4,304,818.56	(58,945)
70– < 80 years old	3,037,453.17	(53,463)	2,505,2267.45	(65,383)
≥80 years old	1,886,302.14	(42,544)	1,063,426.27	(37,993)
Metabolic disorder				
None	25,315,272.16	(140,232)	25,547,307.25	(126,335)
Diabetes only	332,728.06	(3,454)	380,158.52	(4,785)
Hypertension only	2,992,067.24	(42,711)	2,039,554.10	(36,922)
Hypercholesterolemia only	571,379.62	(6,742)	633,138.95	(7,949)
Diabetes and hypertension	200,656.41	(3,509)	214,462.59	(4,495)
Diabetes and hypercholesterolemia	93,514.99	(1,158)	135,247.58	(1,614)
Hypertension and hypercholesterolemia	929,262.47	(14,361)	985,342.21	(18,460)
Diabetes, hypertension and hypercholesterolemia	273,576.23	(4,639)	346,818.08	(6,576)

**Fig. 1:**
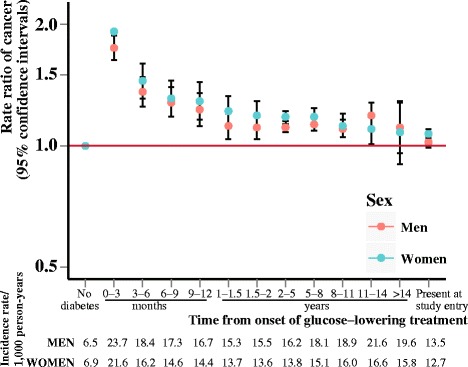
Rate ratios of cancer according to duration of diabetes. Incidence rate ratios of all-site cancer risk according to duration of diabetes, adjusted for age and calendar year and stratified by sex

**Fig. 2:**
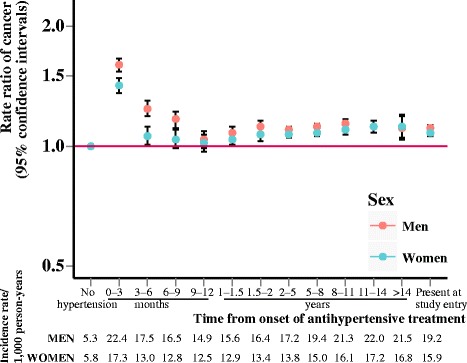
Rate ratios of cancer according to duration of hypertension. Incidence rate ratios of all-site cancer risk according to duration of hypertension, adjusted for age and calendar year and stratified by sex

**Fig. 3:**
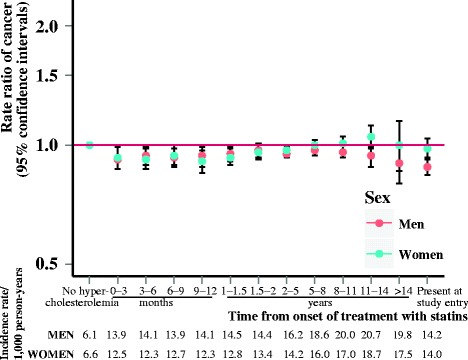
Rate ratios of cancer according to duration of hypercholesterolemia. Incidence rate ratios of all-site cancer risk according to duration of hypercholesterolemia, adjusted for age and calendar year and stratified by sex

We observed that diabetes (Fig. 1) and hypertension (Fig. 2) were associated with increased risk of cancer throughout follow-up, whereas hypercholesterolemia (Fig. 3) over follow-up was associated with decreased or not significantly associated with cancer risk (IRRs and incidence rates illustrated in Additional file 1: Figure S1). Risk of being diagnosed with cancer was highest immediately after diagnosis of diabetes or hypertension, but stabilized after about one year of follow-up. Diabetes appeared to be associated with the highest relative risks of cancer.

**Fig. 4:**
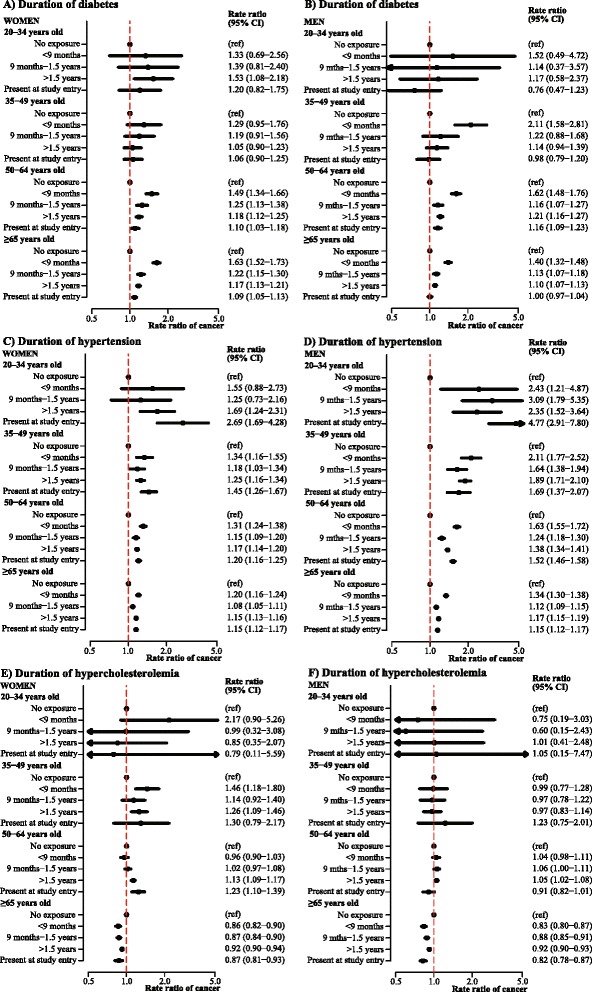
Rate ratios of cancer in women and men according to duration of disorder, stratified by age groups. Incidence rate ratios of all-site cancer according to duration of **a** diabetes in women, **b** diabetes in men, **c** hypertension in women, **d** hypertension in men, **e** hypercholesterolemia in women and **f** hypercholesterolemia in men adjusted for age and calendar year, and stratified by age groups. CI, confidence interval

The effect of diabetes and hypertension on cancer risk differed by age (P for interaction <0.001). The age-stratified analyses showed that the highest relative risks of cancer for both men and women were observed in the younger age groups (Fig. 4a-f).

In women, diabetes was associated with increased risk of kidney and hepatic cancer, and strongly associated with endometrial and pancreatic cancer risk (Additional file 1: Figure S2A), whereas hypertension in women was associated with increased risk of kidney and hepatic cancer, and we observed a trend of increased risk of gall bladder cancer (Additional file 1: Figure S2C). In men, diabetes as well as hypertension was associated with increased risk of hepatic, kidney and colorectal cancer (Additional file 1: Figure S2B, D), and as in women, diabetes was associated with particularly high risk of pancreatic cancer (Additional file 1: Figure S2B). Additionally, hypertension in men was associated with increased risk of prostate cancer (Additional file 1: Figure S2D). Hypercholesterolemia was not associated with subtypes of cancer in women (Additional file 1: Figure S2E) or men (Additional file 1: Figure S2F).

### Other analyses

In sensitivity analysis where we disregarded all prescriptions for metformin to women between 20 and 39 years old, although attenuated, cancer risk remained significantly increased throughout follow-up with the IRR in women with diabetes stabilizing around 1.15 after one year of follow-up (results not shown).

Adjustment for educational level did not attenuate the associations between metabolic disorders and cancer (*n* = 4,223,006 with *n* = 408,353 cancer events), and the variable was not retained in the final models.

Due to significant interaction between metabolic disorders and calendar year, we conducted a sensitivity analysis stratified by calendar year intervals (Additional file 1: Table S2). For diabetes, we found a tendency towards decreased risk over the calendar year periods in men, but not in women, however; the CIs were overlapping in both sexes. For hypertension and hypercholesterolemia, there were largely unaltered risks and overlapping CIs over time in both sexes.

## Discussion

The key message of this paper was that being diagnosed with diabetes and hypertension was associated with increased risk of being diagnosed with cancer, whereas hypercholesterolemia (defined as treatment with statins) over the course of follow-up was associated with decreased or neutral cancer risk. The relative risk of cancer was particularly high among young adults with metabolic dysfunction and this risk declined steadily with higher age. Given the observational nature of the data, it is not possible to study causality.

However, the associations could be caused by 1) reverse causality due to undiagnosed cancer prior to diagnosis of a metabolic disorder; 2) the metabolic disorder itself increasing the risk of cancer; 3) the risk factors increasing the risk of developing a metabolic disorder also increase the risk of cancers, thus confounding by unmeasured risk factors most importantly life style factors; or 4) simply being in contact with the health care system increases the risk of a cancer being identified.

Our results showing elevated cancer risk associated with diabetes are consistent with earlier findings [20–23], and our findings were of the same magnitude of previous findings [12, 23]. A Swedish cohort study from 1991 observed that over 20 years of follow-up, diabetes was associated with an increased cancer risk in women with RR 1.1 (95 % CI = 1.0-1.1), but not in men, as well as elevated risks of several cancer types, including pancreatic, primary liver and endometrial cancer [23]. This is also in accordance with our findings with the relative risk of endometrial cancer being particularly high in women <50 years old compared with women >50 years old (IRR for >1.5 years duration of diabetes was 3.94 [95 % CI 2.33-6.70] and 1.81 [95 % CI 1.64-2.00], respectively). More recently, a study from the Me-Can project of six cohorts from Norway, Sweden and Austria reported a cancer risk per 1 mmol/L increment of glucose of 1.05 (95 % CI = 1.01-1.10) in men and 1.11 (95 % CI = 1.05-1.16) in women, for incident all-site cancer [12]. There are several proposed mechanisms underlying the association between diabetes and cancer [24], but increasing evidence points to hyperinsulinemia [25], hyperglycemia [26], obesity, and chronic low-grade inflammation [27–30]. In this context, obesity is of particular importance, because adipose tissue is an endocrine organ known to produce several adipokines that modulate inflammation and insulin resistance [27, 28]. Approximately 47 % of Danish residents over 16 years of age are overweight and 13 % are obese [31, 32], and since about 80 % of patients with type 2 diabetes are overweight or obese [33], a substantial number of individuals with diabetes in our study can be assumed to be obese. Obesity is a pro-inflammatory state, and although the association between hyperglycemia and overall cancer incidence has been observed to remain after adjustment for BMI [12], and obesity and hyperglycemia increased cancer risk synergistically in the Framingham cohort [11], the causal relation is complex and not fully understood.

We observed hypertension to be associated with significantly increased cancer risks, and although previous studies on the association between hypertension and cancer risk are sparse, our finding of a stronger association in men was in accordance with an observational cohort study based on health examinations comprising nearly 580,000 adults. The study found elevated systolic blood pressure to be associated with an increase in risk of total incident cancer among men (hazard ratio 1.17 [95 % CI = 1.10-1.23] in men, but a non-statistically significant 6 % increase [95 % CI 0.99-1.14] in women) [13].

We observed that the risk of being diagnosed with a cancer diagnosis was significantly higher following initiation of medical treatment for a metabolic disorder, which we interpret as a situation of surveillance bias, as individuals with a known metabolic disorder may have more frequent contact with health professionals and thereby a higher likelihood of having other metabolic disorders or preclinical cancers detected. However, even in the long-term we found diabetes and hypertension to be associated with increased cancer risk. These findings are in line with previous studies [34–37], including a Danish study exploring cancer risk according to time elapsed since treatment initiation with specific types of glucose-lowering medications [38]. However, since cancer (overt or occult) is diabetogenic, part of the association between diabetes and cancer could be reverse causality as i.e. circulating cytokines in cancer may affect glucose metabolism directly. Furthermore, both cancer and diabetes can stay subclinical for years, making it a case of the chicken and the egg.

In this study, hypercholesterolemia defined as initiation of treatment with statins, was associated with decreased or neutral cancer risk in both men and women. Statins are by far the most commonly used group of lipid medications, prescribed for elevated total-cholesterol and LDL-cholesterol, and their use has been associated with reduced risk of incident cancer in several, but not all, studies [39–41].

The absolute cancer incidence in young individuals were low, albeit far from nonexisting, and 4.1 % (*n* = 16,755) of the cancer events in this study were detected in individuals <40 years of age (3.7 % when excluding cervix cancers). Previous studies on associations between diabetes, hypertension and hypercholesterolemia, and overall as well as site-specific cancer, are limited, and given the low absolute cancer incidence rate, a large-scale study is needed to conduct reliable analyses. The most prevalent cancer types also differ between younger and older age-groups [42]. Young adults diagnosed with cancer thus constitute a selected population with a high risk factor burden. In light of the growing obesity problem worldwide, which also influences the risk of diabetes, hypertension and hypercholesterolemia, it is important to be aware of any associations, since acknowledgement hereof may increase the likelihood of early cancer detection.

### Strengths and limitations

The major strengths of this nationwide register-based study are the completeness of the records of hospital diagnoses and prescription claims; the minimal risk of selection bias and loss to follow-up; and the large population of both men and women, which provide sufficient power to conduct detailed analyses even in low-risk subgroups. Nonetheless, our study has some important limitations, primarily that the definition of metabolic disorders primarily relied on claimed prescriptions, and the true number of people diagnosed with these metabolic disorders may therefore be underestimated [43]. This study focused on diabetes requiring treatment with a glucose-lowering drug. During the later part of the study period it was very uncommon to use diet alone and therefore we consider this definition adequate to identify the vast majority of patients with diabetes, and according to a study by Carstensen et al. identification of diabetes patients by glucose-lowering drugs in the Danish registries have a sensitivity of 72 %, and a positive predictive value of 95 % [18]. There is a risk of overdiagnosing patients with polycystic ovary syndrome, but we consider this problem minor. In a recent study a total of 19,195 cases were identified in Denmark between 1995 and 2012 of which only 10 % received metformin [44]. Further, this condition is known to be associated with an increased risk of diabetes and insulin resistance. We also conducted a sensitivity analysis excluding females given metformin between the ages of 20 and 39, which yielded similar results. We did not exclude gestational diabetes requiring insulin, since these women have a high risk of later developing diabetes. Lastly for diabetes, the vast majority of individuals with diabetes have type 2 diabetes, which is often treated with metformin initially; a drug linked to decreased cancer incidence in recent years [24], and our findings may therefore be an underestimation of the true risk.

For hypertension, 23.3 % of our study population was defined as having hypertension either at study entry or developed during follow-up, which is comparable to the age-adjusted prevalence of 22.3 % found by a comprehensive clinical study of Danish adults aged 20-89 [45]. Angiotensin receptor blockers and angiotensin converting enzyme inhibitors are two of the most commonly used antihypertensive agents. Although study findings are conflicting regarding their relation to cancer risk [46-49], the majority of studies conclude that antihypertensive agents do not initiate cancer development [37, 47, 50], and recent studies even suggest a possible inverse association with cancer incidence [48, 51].

Cancer events were primarily identified from DNPR with supplement from the Danish Register of Causes of Death. We did not have access to The Danish Cancer Registry, which primarily gathers information from DNPR, in addition to the Danish Register of Causes of Death, and finally, from the Danish Pathology Registry. Since we do not have access to the pathology registry, our study may underestimate the number of cancer incidences, although the number is considered low, since the Danish Pathology Registry forward information to the Danish Register of Causes of Death.

Data on anthropometric measures, life style habits and smoking are unfortunately not available from the Danish nationwide registers. Presence of metabolic disorders may be a marker of obesity, poor diet, physical inactivity and smoking, all of which are known to increase the risk of cancer, i.e. smoking strongly increases the risk of several cancers, particularly lung cancer and cancer of the urinary bladder. Socioeconomic status is correlated to life style factors such as obesity and smoking, and although only an approximation, our results were not altered by adjustment for socioeconomic status.

## Conclusions

In this large cohort of more than 4.6 million men and women of ages 20–89, we found that diabetes and hypertension were associated with an increased risk of incident all-site cancer across sex, age and calendar year with particularly high relative risks in the youngest age groups. Diabetes was associated with the highest cancer risk in both sexes, followed by hypertension, whereas hypercholesterolemia treated with statins was not associated with cancer risk. Our results stress the importance of prophylactic awareness among patients with diabetes and/or hypertension, not only because of increased cardiovascular risk, but also because of increased associated cancer risk.

## Additional file

Additional file 1:
**Supplemental material, Berger** ***et al.*** (PDF 331 kb)

## Abbreviations

SD: standard deviation
IR: incidence rate
IRR: incidence rate ratio
PYs: person years
CI: confidence interval
ICD: International classification of diseases
ATC: Anatomical Therapeutic Chemical (classification system)
P: probability
BMI: body mass index
LDL: low-density lipoprotein
HDL: high-density lipoprotein
