# Supplementary material for: Associations between metabolic disorders and risk of cancer in Danish men and women – a nationwide cohort study
Source: BMC Cancer. 2016 Feb 22;16:133. doi: 10.1186/s12885-016-2122-7 (PMC4762170; doi:10.1186/s12885-016-2122-7)

SUPPLEMENTAL MATERIAL

TABLE S1. Population characteristics at study entry

TABLE S2. Rate ratios of cancer associated with diabetes, hypertension and hypercholesterolemia by calendar year intervals

FIGURE S1. Rate ratios of cancer associated with diabetes, hypertension and hypercholesterolemia according to duration of metabolic disorder

FIGURE S2. Rate ratios of subtypes of cancer associated with diabetes, hypertension and hypercholesterolemia according to duration of metabolic disorder

TABLE S1: The study population at study entry

Variable	Men	Women	Total
Population, *n* (%)	2,401,193 (49.8)	2,424,949 (50.2)	4,826,142 (100)
Age (mean years) (SD)	40.3 (18.1)	42.4 (19.6)	41.4 (18.9)
Follow-up (mean years) (SD)	12.6 (5.8)	12.6 (5.7)	12.6 (5.7)
Diabetes, *n* (%)	34,548 (1.4)	31,142 (1.3)	65,690 (1.4)
Hypertension, *n* (%)	64,378 (2.7)	89,238 (3.7)	153,616 (3.2)
Dyslipidemia (statins), *n* (%)	8,928 (0.4)	6,095 (0.3)	15,023 (0.3)

TABLE S2A. IRRs (95%CI) of all-site cancer in women according to duration of each metabolic disorder and calendar year, and adjusted for age*

	**1996-1999**	**2000-2003**	**2004-2007**	**2008-2012**
**Duration of diabetes**				
**No diabetes**	(ref.)	(ref.)	(ref.)	(ref.)
**0-<9 mths**	1.49 (1.30-1.70)	1.64 (1.45-1.85)	1.56 (1.40-1.75)	1.53 (1.40-1.67)
**9 mths–1.5 years**	1.15 (1.00-1.32)	1.26 (1.13-1.41)	1.29 (1.17-1.42)	1.16 (1.07-1.26)
**>1.5 years**	0.94 (0.79-1.12)	1.13 (1.06-1.21)	1.16 (1.10-1.22)	1.17 (1.13-1.21)
**Prevalent at study entry**	1.14 (1.08-1.20)	1.04 (0.98-1.10)	1.08 (1.01-1.16)	1.01 (0.93-1.09)
**Duration of hypertension**				
**No hypertension**	(ref.)	(ref.)	(ref.)	(ref.)
**0-<9 mths**	1.10 (1.03-1.17)	1.09 (1.03-1.15)	1.19 (1.13-1.25)	1.28 (1.22-1.35)
**9 mths–1.5 years**	1.08 (1.02-1.14)	1.02 (0.97-1.07)	1.00 (0.96-1.05)	1.07 (1.02-1.11)
**>1.5 years**	0.95 (0.89-1.02)	1.06 (1.03-1.09)	1.05 (1.02-1.07)	1.11 (1.09-1.13)
**Prevalent at study entry**	1.06 (1.03-1.10)	1.06 (1.03-1.10)	1.07 (1.03-1.11)	1.14 (1.09-1.18)
**Duration of hypercholesterolemia**				
**No hyper-cholesterolemia**	(ref.)	(ref.)	(ref.)	(ref.)
**0-<9 mths**	0.87 (0.73-1.03)	0.88 (0.80-0.97)	0.88 (0.83-0.94)	0.98 (0.93-1.03)
**9 mths–1.5 years**	0.94 (0.81-1.11)	0.82 (0.75-0.90)	0.92 (0.87-0.96)	0.98 (0.94-1.02)
**>1.5 years**	0.85 (0.69-1.03)	1.00 (0.95-1.07)	0.96 (0.93-0.99)	0.97 (0.95-0.99)
**Prevalent at study entry**	0.99 (0.88-1.11)	0.94 (0.84-1.06)	1.03 (0.92-1.16)	0.95 (0.84-1.07)

*All results arise from the same model.

IRR, incidence rate radio; mths, months; 95%CI, 95% confidence interval: ref., reference

TABLE S2B. IRRs (95%CI) of all-site cancer in men according to duration of each metabolic disorder and calendar year intervals, and adjusted for age*

	**1996-1999**	**2000-2003**	**2004-2007**	**2008-2012**
**Duration of diabetes**				
**No diabetes**	(ref.)	(ref.)	(ref.)	(ref.)
**0-<9 mths**	1.40 (1.24-1.58)	1.66 (1.50-1.84)	1.50 (1.37-1.64)	1.34 (1.24-1.44)
**9 mths–1.5 years**	1.05 (0.93-1.19)	1.11 (1.00-1.22)	1.13 (1.03-1.22)	1.14 (1.06-1.22)
**>1.5 years**	1.34 (1.17-1.52)	1.15 (1.08-1.21)	1.11 (1.06-1.15)	1.09 (1.06-1.12)
**Prevalent at study entry**	1.10 (1.05-1.16)	1.05 (0.99-1.11)	0.94 (0.88-1.01)	0.92 (0.86-0.99)
**Duration of hypertension**				
**No hypertension**	(ref.)	(ref.)	(ref.)	(ref.)
**0-<9 mths**	1.30 (1.22-1.37)	1.32 (1.26-1.40)	1.31 (1.25-1.37)	1.38 (1.31-1.44)
**9 mths–1.5 years**	1.08 (1.02-1.14)	1.04 (0.99-1.09)	1.08 (1.04-1.13)	1.11 (1.06-1.15)
**>1.5 years**	1.14 (1.06-1.21)	1.07 (1.04-1.10)	1.11 (1.08-1.13)	1.14 (1.11-1.16)
**Prevalent at study entry**	1.08 (1.04-1.12)	1.08 (1.04-1.12)	1.12 (1.07-1.16)	1.21 (1.16-1.26)
**Duration of hypercholesterolemia**				
**No hyper-cholesterolemia**	(ref.)	(ref.)	(ref.)	(ref.)
**0-<9 mths**	0.78 (0.66-0.91)	0.85 (0.78-0.93)	0.93 (0.88-0.98)	0.96 (0.91-1.01)
**9 mths–1.5 years**	1.00 (0.87-1.14)	0.87 (0.80-0.94)	0.92 (0.88-0.96)	1.00 (0.96-1.04)
**>1.5 years**	0.76 (0.63-0.91)	0.88 (0.84-0.93)	0.95 (0.92-0.98)	0.96 (0.94-0.98)
**Prevalent at study entry**	0.75 (0.67-0.85)	0.92 (0.84-1.01)	0.92 (0.84-1.01)	0.90 (0.82-0.99)

*All results arise from the same model.

IRR, incidence rate radio; mths, months; 95%CI, 95% confidence interval: ref., reference

FIGURE S1. Rate ratios of cancer associated with A) diabetes, B) hypertension and C) hypercholesterolemia according to duration of metabolic disorder, stratified by sex and adjusted for age.

A) Rate ratios of cancer according to duration of diabetes

Duration of diabetes	Rate ratio (95% CI)	IR/1,000 PYs (95% CI)
**Women**		
None	(ref)	6.8 (6.8−6.9)
0−3 mths	1.92 (1.77−2.09)	21.6 (19.9−23.5)
>3−6 mths	1.45 (1.31−1.60)	16.2 (14.7−17.8)
>6−9 mths	1.31 (1.18−1.45)	14.6 (13.1−16.1)
>9−12 mths	1.29 (1.16−1.44)	14.4 (12.9−16.0)
>1−1.5 yrs	1.22 (1.13−1.33)	13.7 (12.7−14.9)
>1.5−2 yrs	1.19 (1.10−1.29)	13.6 (12.5−14.7)
>2−5 yrs	1.18 (1.13−1.22)	13.8 (13.3−14.4)
>5−8 yrs	1.18 (1.12−1.24)	15.1 (14.4−15.8)
>8−11 yrs	1.12 (1.05−1.20)	15.9 (15.0−17.0)
>11−14 yrs	1.10 (1.01−1.20)	16.6 (15.2−18.1)
>14 yrs	1.08 (0.90−1.29)	15.8 (13.3−18.8)
Baseline	1.07 (1.04−1.10)	12.7 (12.3−13.1)
**Men**		
None	(ref)	6.5 (4.5−6.5)
0−3 mths	1.75 (1.63−1.88)	23.7 (22.0−25.4)
>3−6 mths	1.36 (1.25−1.48)	18.4 (16.9−20.0)
>6−9 mths	1.28 (1.18−1.40)	17.3 (15.9−18.9)
>9−12 mths	1.23 (1.12−1.35)	16.7 (15.2−18.2)
>1−1.5 yrs	1.12 (1.04−1.20)	15.2 (14.2−16.3)
>1.5−2 yrs	1.11 (1.04−1.19)	15.5 (14.4−16.6)
>2−5 yrs	1.11 (1.08−1.15)	16.2 (15.7−16.8)
>5−8 yrs	1.13 (1.09−1.18)	18.1 (17.4−18.8)
>8−11 yrs	1.10 (1.05−1.16)	18.9 (18.0−19.9)
>11−14 yrs	1.19 (1.11−1.28)	21.6 (20.1−23.1)
>14 yrs	1.11 (0.96−1.28)	19.6 (17.1−22.6)
Baseline	1.02 (0.99−1.05)	13.5 (13.1−13.9)

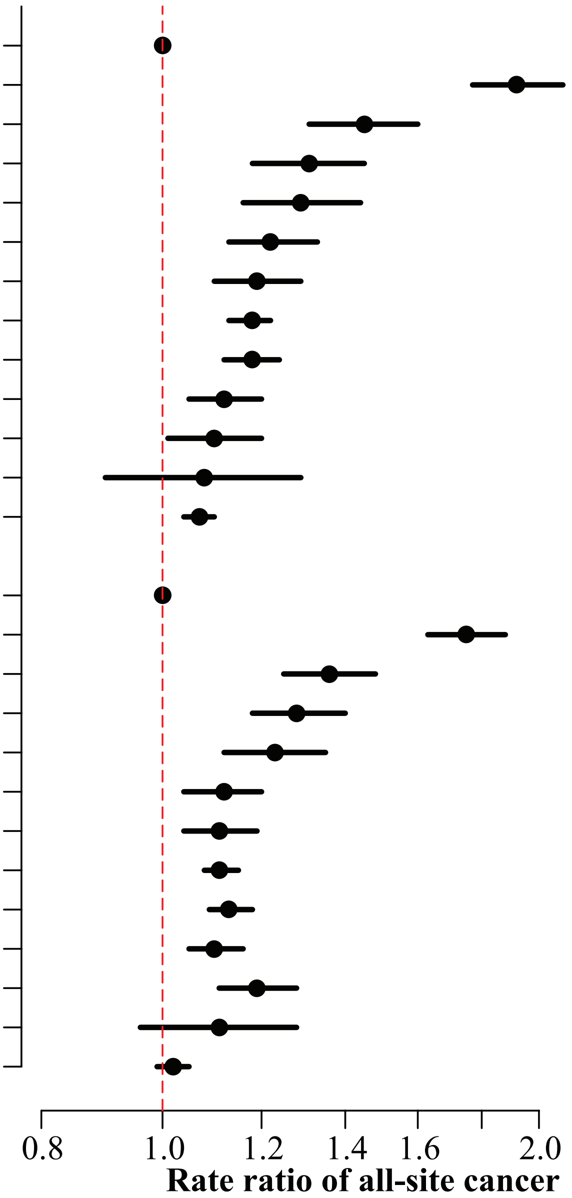

B) Rate ratios of cancer according to duration of hypertension

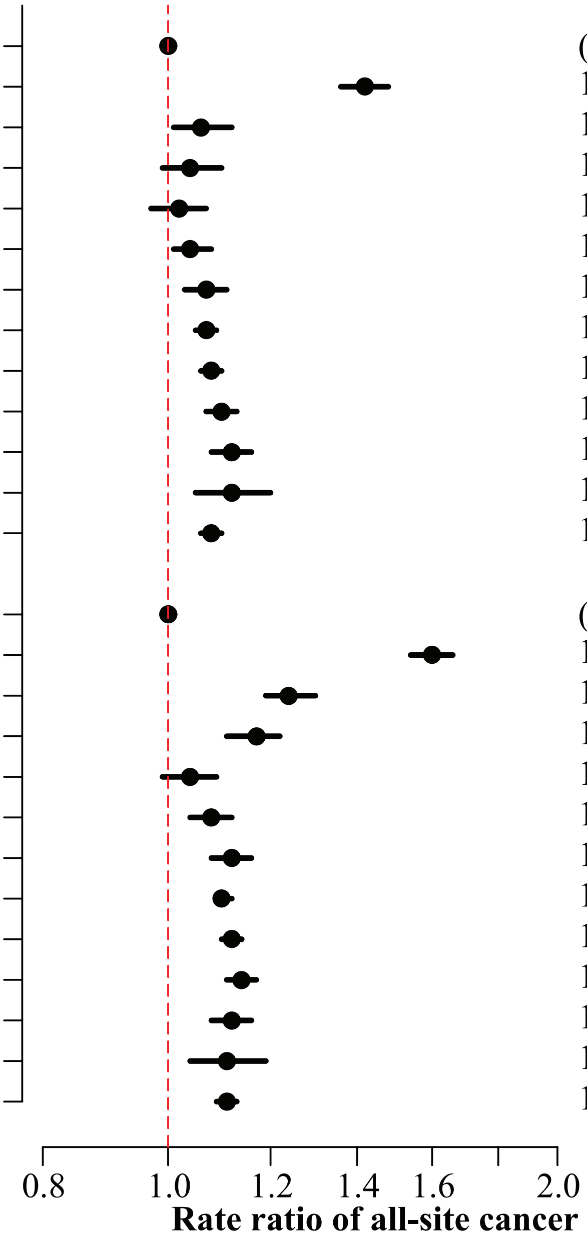

Duration of hypertension	Rate ratio (95% CI)	IR/1,000 PYs (95% CI)
**Women**		
None	(ref)	5.8 (5.7−5.8)
0−3 mths	1.42 (1.36−1.48)	17.3 (16.6−18.0)
>3−6 mths	1.06 (1.01−1.12)	13.0 (12.3−13.6)
>6−9 mths	1.04 (0.99−1.10)	12.8 (12.2−13.4)
>9−12 mths	1.02 (0.97−1.07)	12.5 (11.9−13.2)
>1−1.5 yrs	1.04 (1.01−1.08)	12.9 (12.4−13.4)
>1.5−2 yrs	1.07 (1.03−1.11)	13.3 (12.9−13.8)
>2−5 yrs	1.07 (1.05−1.09)	13.8 (13.6−14.0)
>5−8 yrs	1.08 (1.06−1.10)	15.0 (14.8−15.3)
>8−11 yrs	1.10 (1.07−1.13)	16.1 (15.7−16.5)
>11−14 yrs	1.12 (1.08−1.16)	17.2 (16.6−17.8)
>14 yrs	1.12 (1.05−1.20)	16.8 (15.8−17.9)
Baseline	1.08 (1.06−1.10)	15.9 (15.7−16.2)
**Men**		
None	(ref)	5.3 (5.2−5.3)
0−3 mths	1.60 (1.54−1.66)	22.4 (21.6−23.3)
>3−6 mths	1.24 (1.19−1.30)	17.5 (16.8−18.3)
>6−9 mths	1.17 (1.11−1.22)	16.5 (15.8−17.3)
>9−12 mths	1.04 (0.99−1.09)	14.9 (14.2−15.6)
>1−1.5 yrs	1.08 (1.04−1.12)	15.6 (15.0−16.1)
>1.5−2 yrs	1.12 (1.08−1.16)	16.4 (15.9−17.0)
>2−5 yrs	1.10 (1.09−1.12)	17.2 (16.9−17.4)
>5−8 yrs	1.12 (1.10−1.14)	19.4 (19.1−19.8)
>8−11 yrs	1.14 (1.11−1.17)	21.3 (20.8−21.8)
>11−14 yrs	1.12 (1.08−1.16)	22.0 (21.2−22.7)
>14 yrs	1.11 (1.04−1.19)	21.5 (20.1−22.9)
Baseline	1.11 (1.09−1.13)	19.2 (18.9−19.5)

C) Rate ratios of cancer according to duration of hypercholesterolemia

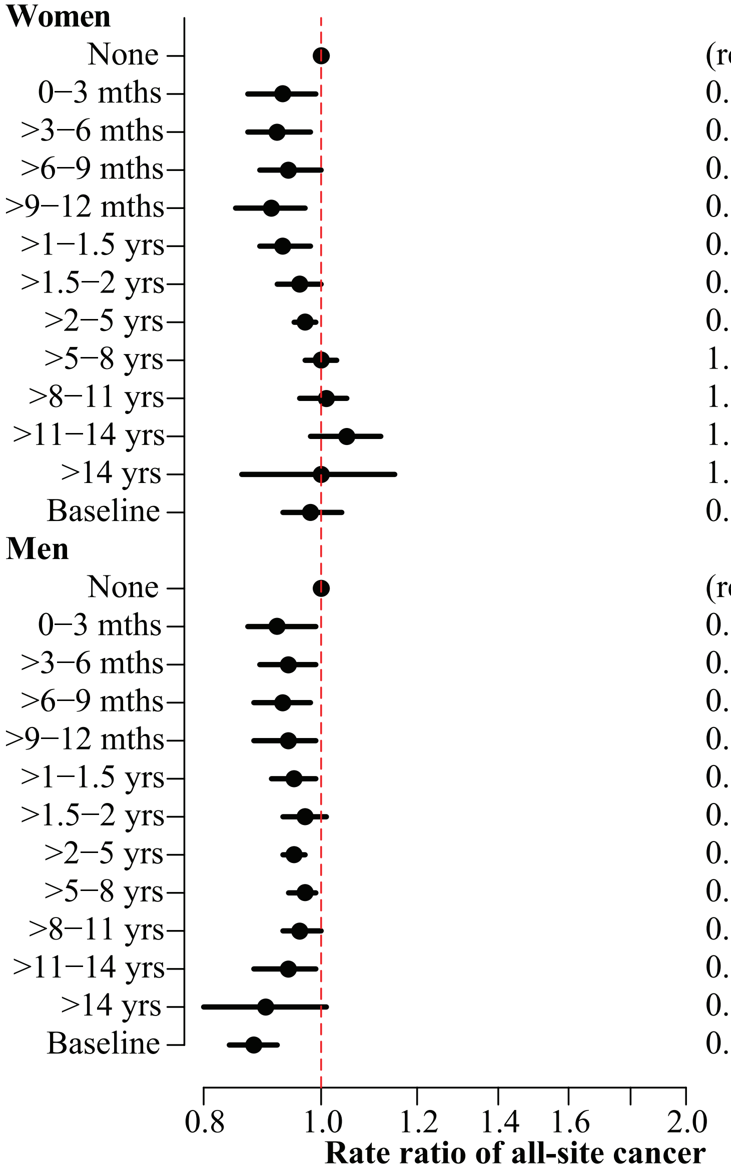

Duration of hypertension	Rate ratio (95% CI)	IR/1,000 PYs (95% CI)
**Women**		
None	(ref)	6.6 (6.6−6.6)
0−3 mths	0.93 (0.87−0.99)	12.5 (11.7−13.3)
>3−6 mths	0.92 (0.87−0.98)	12.3 (11.6−13.1)
>6−9 mths	0.94 (0.89−1.00)	12.7 (11.9−13.5)
>9−12 mths	0.91 (0.85−0.97)	12.3 (11.5−13.1)
>1−1.5 yrs	0.93 (0.89−0.98)	12.8 (12.2−13.4)
>1.5−2 yrs	0.96 (0.92−1.00)	13.4 (12.8−14.0)
>2−5 yrs	0.97 (0.95−0.99)	14.2 (13.9−14.5)
>5−8 yrs	1.00 (0.97−1.03)	16.0 (15.6−16.4)
>8−11 yrs	1.01 (0.96−1.05)	17.0 (16.3−17.7)
>11−14 yrs	1.05 (0.98−1.12)	18.6 (17.5−19.9)
>14 yrs	1.00 (0.86−1.15)	17.5 (15.2−20.2)
Baseline	0.98 (0.93−1.04)	14.0 (13.2−14.9)
**Men**		
None	(ref)	6.1 (6.1−6.2)
0−3 mths	0.92 (0.87−0.99)	13.9 (13.1−14.7)
>3−6 mths	0.94 (0.89−0.99)	14.1 (13.4−14.9)
>6−9 mths	0.93 (0.88−0.98)	13.9 (13.2−14.8)
>9−12 mths	0.94 (0.88−0.99)	14.1 (13.3−15.0)
>1−1.5 yrs	0.95 (0.91−0.99)	14.5 (13.9−15.2)
>1.5−2 yrs	0.97 (0.93−1.01)	15.4 (14.8−16.0)
>2−5 yrs	0.95 (0.93−0.97)	16.2 (15.9−16.6)
>5−8 yrs	0.97 (0.94−0.99)	18.6 (18.2−19.1)
>8−11 yrs	0.96 (0.93−1.00)	20.0 (19.3−20.7)
>11−14 yrs	0.94 (0.88−0.99)	20.7 (19.6−21.9)
>14 yrs	0.90 (0.80−1.01)	19.8 (17.6−22.2)
Baseline	0.88 (0.84−0.92)	14.2 (13.5−14.9)

FIGURE S2. Rate ratios of subtypes of cancer associated with diabetes, hypertension and hypercholesterolemia according to duration of metabolic disorder

A) Diabetes and risk of subtypes of cancer in women

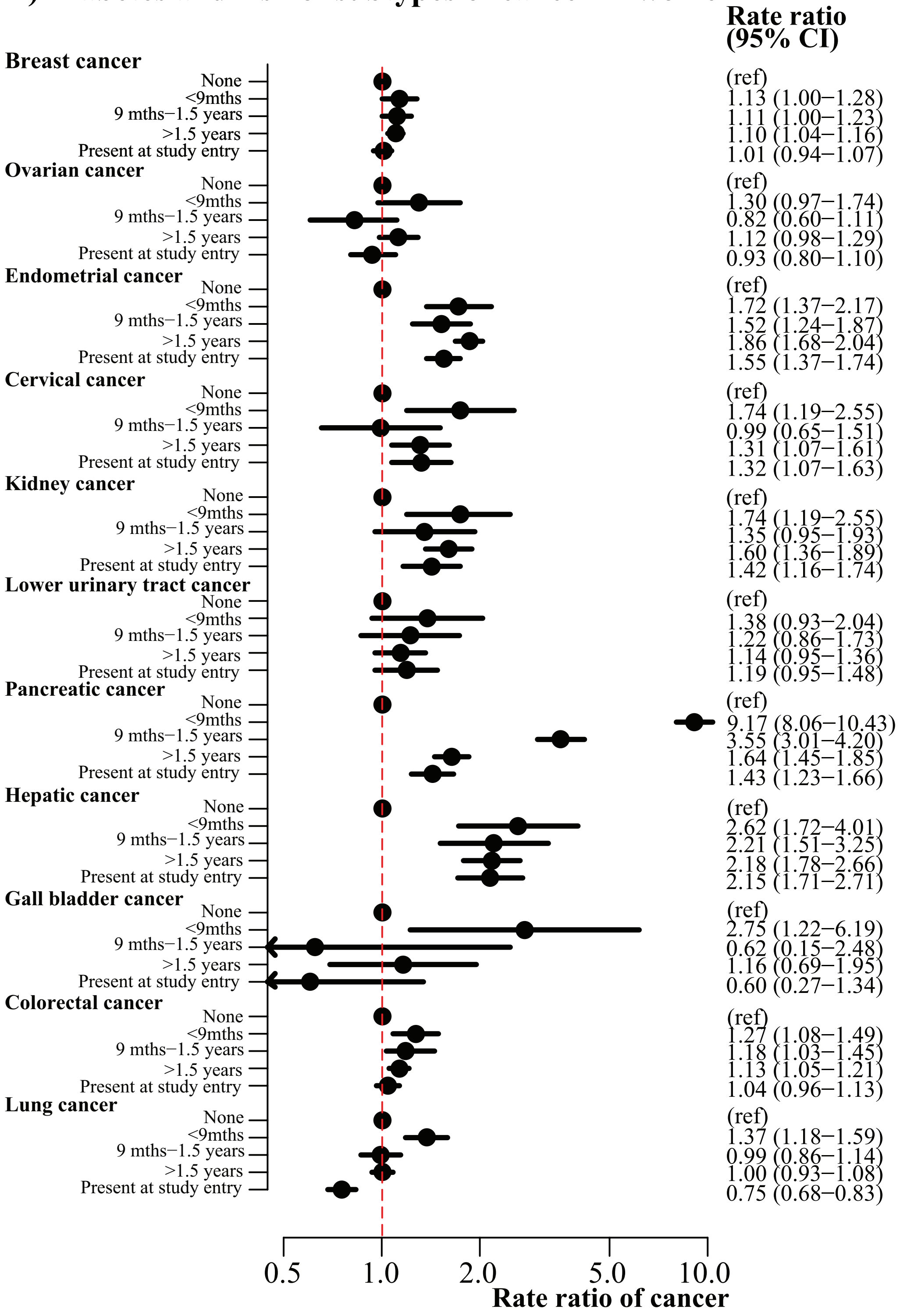

B) Diabetes and risk of subtypes of cancer in men

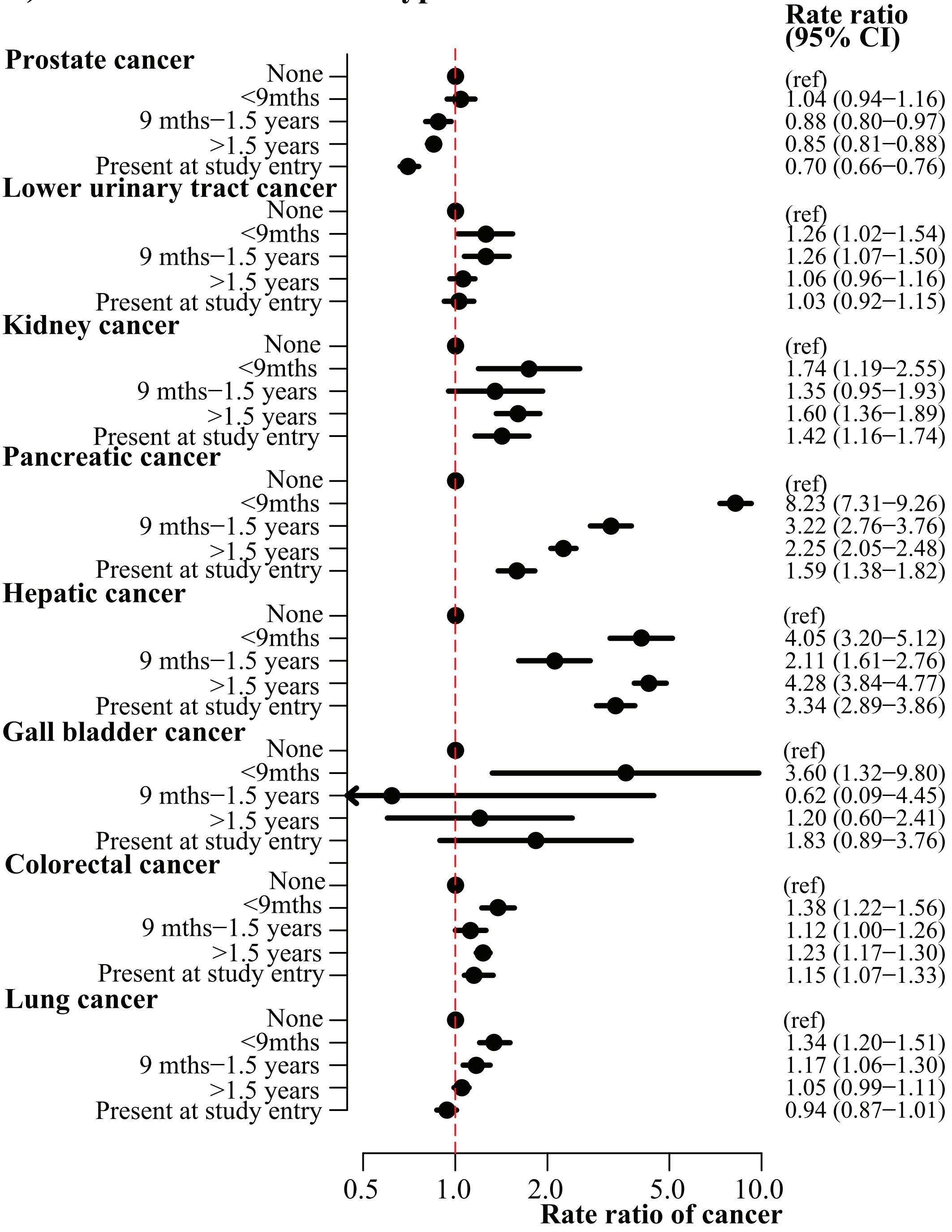

C) Hypertension and risk of subtypes of cancer in women

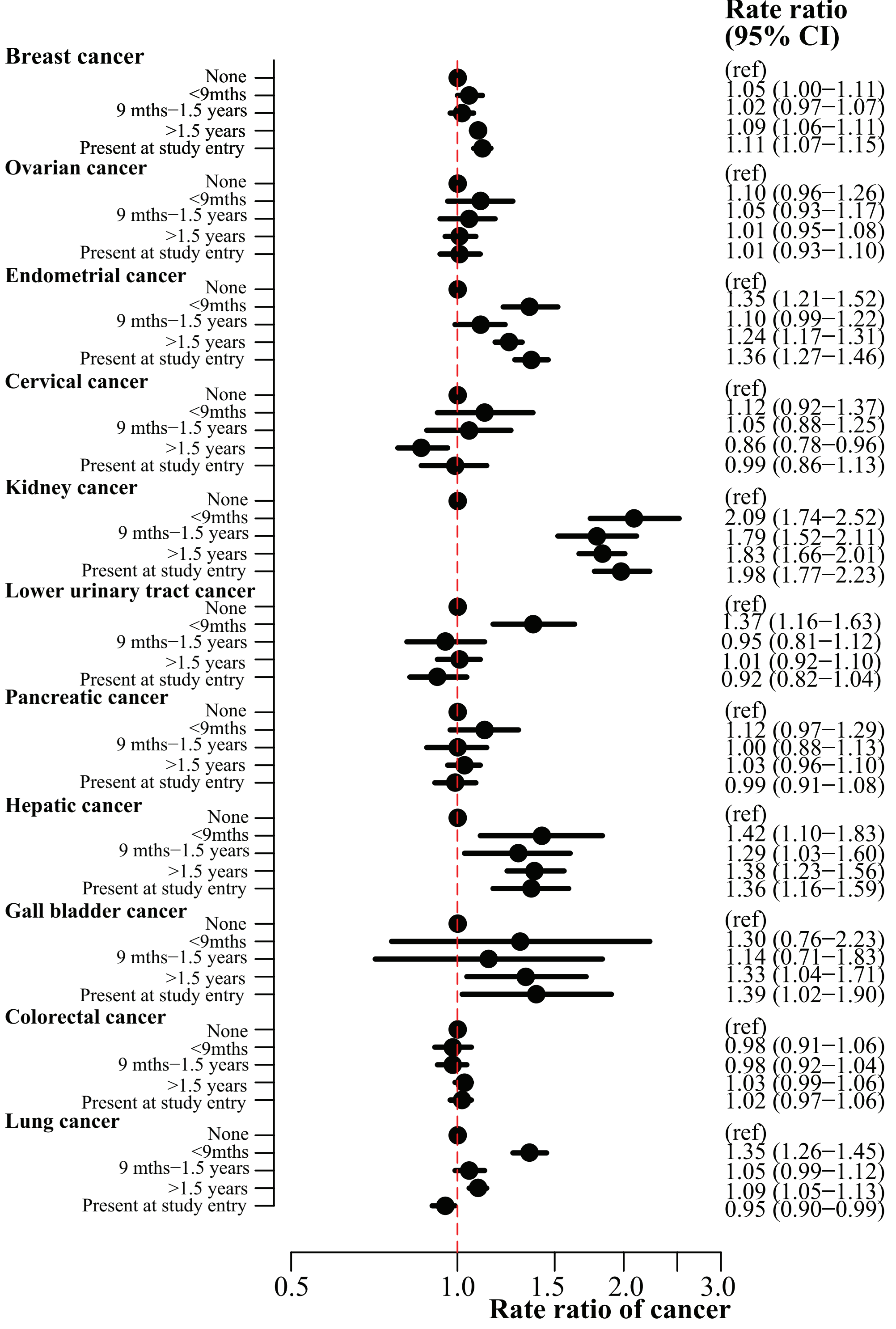

D) Hypertension and risk of subtypes of cancer in men

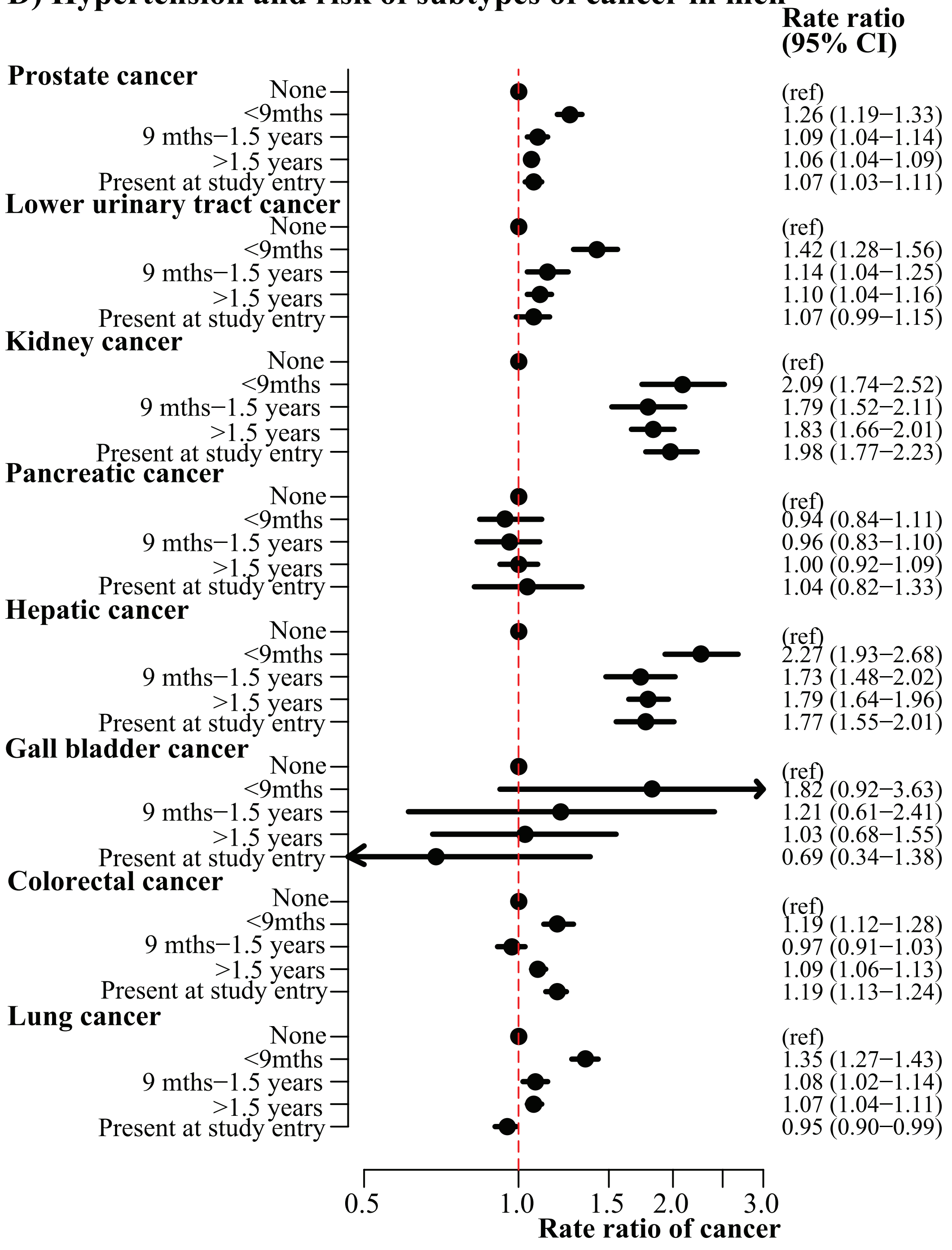

E) Hypercholesterolemia and risk of subtypes of cancer in women

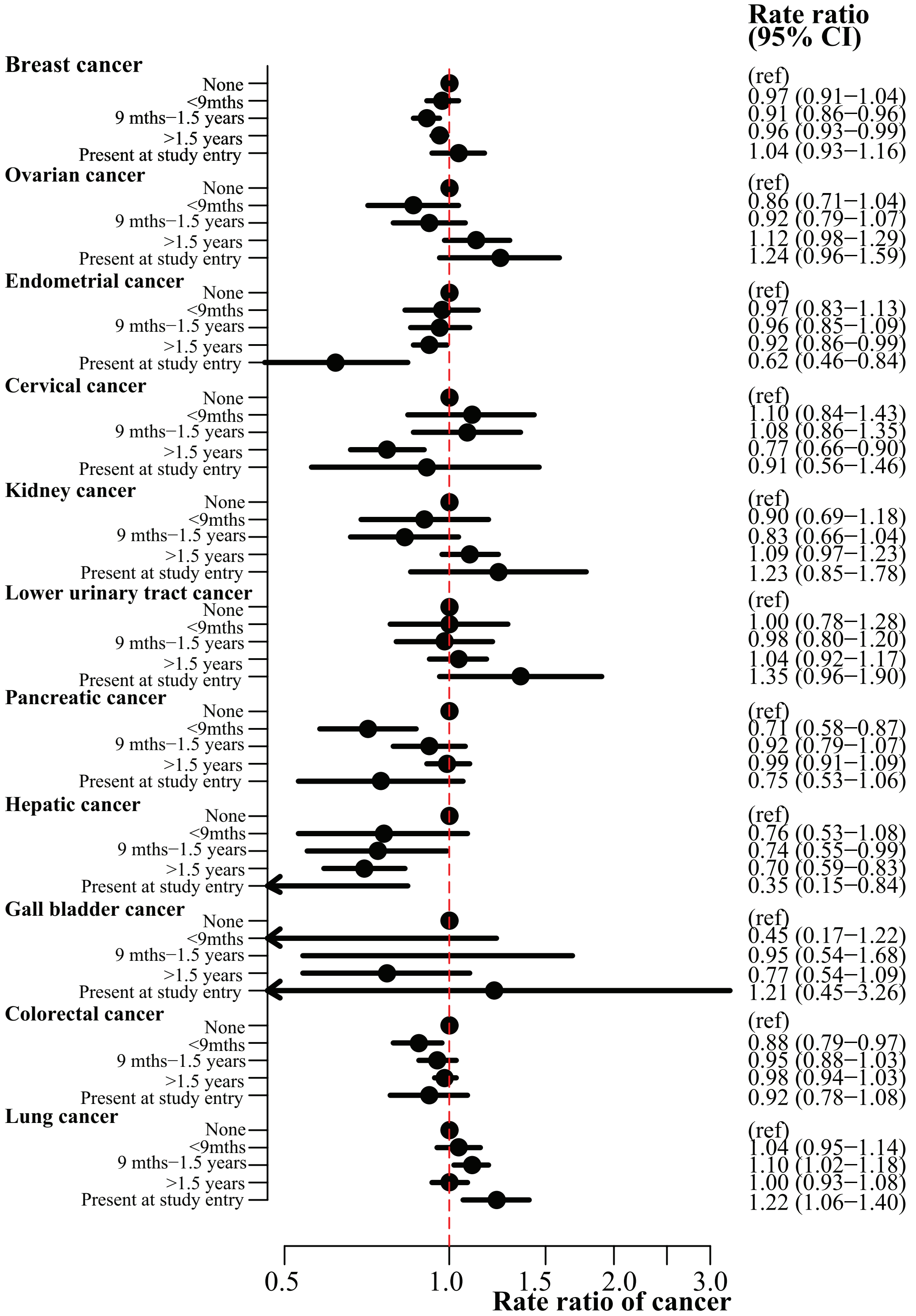

F) Hypercholesterolemia and risk of subtypes of cancer in men

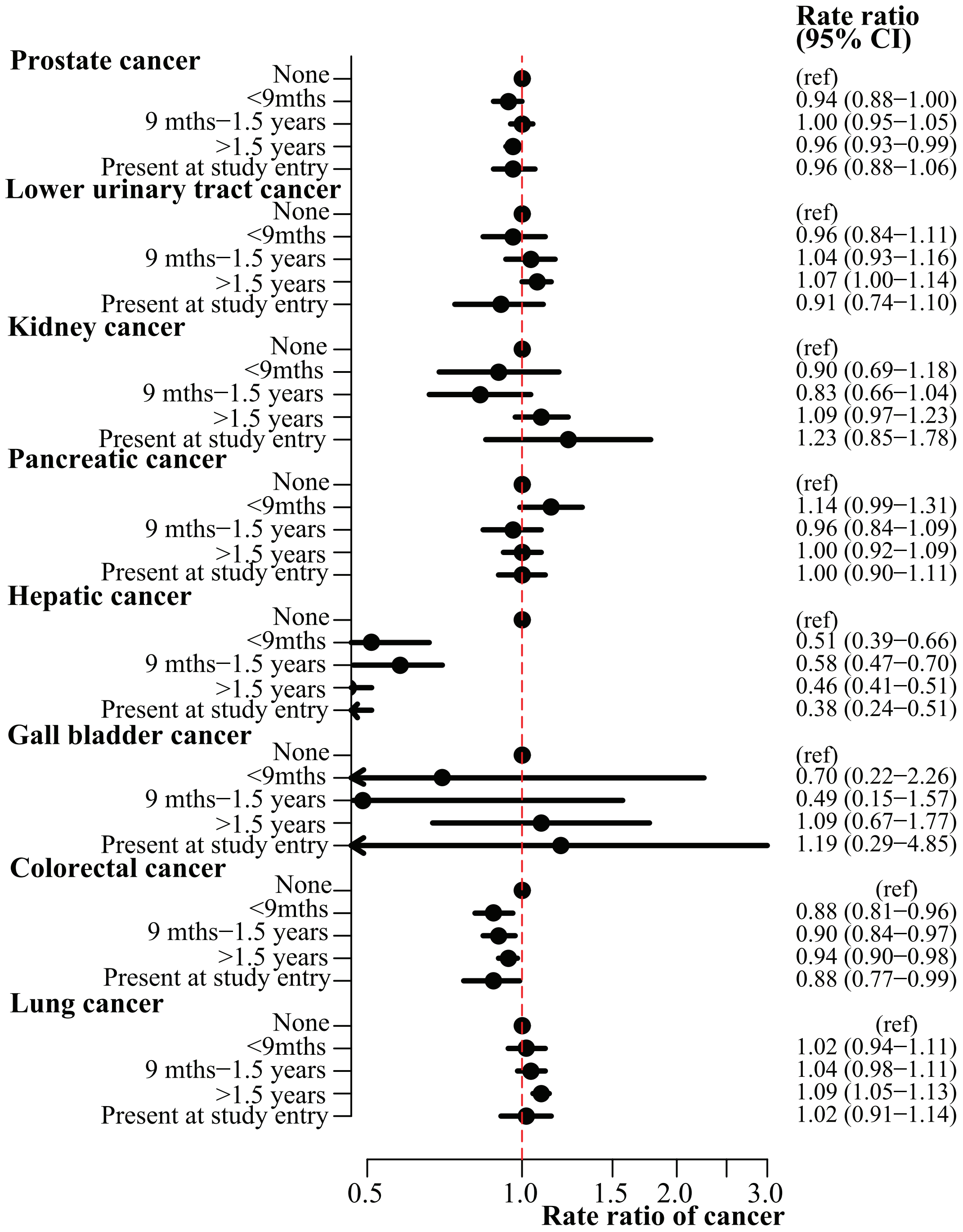

Supplement: Additional file 1: — Supplemental material, Berger et al. (PDF 331 kb) [file 12885_2016_2122_MOESM1_ESM.pdf]
